# Editorial: Harnessing resilience of orphan crops to the effects of drought and heat: A route towards a sustainable agriculture

**DOI:** 10.3389/fpls.2022.1086143

**Published:** 2022-12-09

**Authors:** Diouf D., Wang L., Hickey L. T., Campbell B. C.

**Affiliations:** ^1^ Laboratoire Campus de Biotechnologies Végétales, Département de Biologie Végétale, Faculté des Sciences et Techniques, Université Cheikh Anta Diop, Dakar, Senegal; ^2^ Chinese Academy of Agricultural Sciences-Oil Crops Research Institute (CAAS-OCRI), Wuhan, China; ^3^ Centre for Crop Sciences, Queensland Alliance for Agriculture and Food Innovation, The University of Queensland, Brisbane, QLD, Australia

**Keywords:** drought, heat, orphan crops, breeding, genetics

Orphan crops are critically important in the countries where they are grown, as they are uniquely adapted to the local environmental conditions and eaten as part of regional diets. As such, orphan crops provide an important link in the ongoing challenge of food security in the developing world. A prime example being Africa, whose population currently is estimated at roughly 1.2 billion and could soar to more than 4 billion by 2100 (Vollset et al., 2020). As with the major crops, there are members of all food types, ranging from cereals and legumes, through to vegetables and root and tuber crops. Because they receive less research consideration, the breeding technology for orphan crops is trailing behind the latest in crop improvement expertise. That means that the cultivars farmers plant are less likely to be resilient to drought, flooding, or extreme temperatures, resulting in lower productivity and becoming more vulnerable to pests and disease. Consequently, they produce inferior yields in terms of both quantity and quality.

While the causes of food insecurity are many and varied, the complicating factor of climate change poses additional pressures on farmers, with the combination of severe and prolonged drought and heat waves that can have devastating consequences on small-holder agriculture, economics, and social stability.

In model crops, it is well documented that these abiotic stresses negatively impact plant development, particularly at vegetative and reproductive stages, leading to decreases in yield, yield components and seed micronutrient content. Although, taken individually, the genotypes which are tolerant to drought or heat stress do not necessarily exhibit tolerance to the combination of both these constraints, due to their specific effects on cellular process when they are both acting. Consequently, there is a need to integrate the recent advances performed in physiology, biochemistry, omics, artificial intelligence and micromics technologies to enhance our understanding of the mechanisms that govern tolerance of these abiotic constraints and develop well-adapted climate varieties for improving global food security. In this present e-book, we present a selection of original research and a review aiming to decipher the mechanisms controlling drought or heat tolerance in emerging crops that can play important cultural and food security functions, particularly for low-income earners.

In this regard, the review of Srivastava et al. (2022) which focuses on the achievements and prospects for breeding drought tolerant pearl millet (*Cenchrus americanus*) using conventional and genomics assisted approaches is relevant. They discuss how the recent advancement in high-throughput phenotyping platforms has made it realistic to screen large populations/germplasm for drought adaptive traits. They further elaborate upon the substantial progress that has been made towards the development of genomic resources that have been used to explore genomic variation in pearl millet. Not to mention the use of High-throughput genotyping (HTPG) platforms and Next-generation sequencing (NGS) technology which have helped to provide a greater understanding of germplasm, genomes, genes and markers, and their applications in molecular breeding leading to high-yielding and drought-tolerant pearl millet cultivars.

Further to this review is the original research work detailing the identification of traits associated with terminal drought stress in quinoa (*Chenopodium quinoa*) and Sesame (*Sesamum indicum*) (Zhu et al., 2022; Pandey et al. (2022), In quinoa, it was observed that Calcineurin-like Protein (CBL) and CBL interacting protein kinase (CIPK) were involved in drought regulation. Specifically, the expression levels of CqCIPK11, CqCIPK15, CqCIPK37 and CqCBL13 increased significantly under drought stress. While in sesame, 76 accessions from an Indian core set were used to quantify variation in several traits under irrigated (WW) and terminal drought stress (WS) conditions as well as their association with seed yield over two consecutive years. The range of trait variation among the studied genotypes under WW and WS was significant. Furthermore, the traits associated with seed yield under WW and WS differed, but the overarching discovery was that smaller and cooler canopy genotypes had higher yields.

The e-book also contains several manuscripts detailing the drought response of species such as *Crassocephalum rubens* and *C. crepidioides*, and tea (*Camellia sinensis*) (Adedeji-Badmus et al. 2022; Lu et al. 2022,). *Crassocephalum rubens* and *C. crepidioides* are plant species native to Africa but grow in most tropical and subtropical regions of the world. They are rich in vitamins, minerals, and essential oils and are traditional leafy vegetable and medicinal plants from Sub-Saharan Africa. In this study, Adedeji-Badmus et al. (2022) investigate the seed formation and germination capacities in *C. crepidioides* and *C. rubens*, where it was found that *C. crepidioides* exhibits a higher level of seed dormancy, which could be broken with light, and was correlated with higher amounts of abscisic acid (ABA), a plant hormone that promotes seed dormancy. ABA is also very well-known for its roles in abiotic stress tolerance, and it is shown that tetraploid *C. crepidioides* exhibits a higher level of resistance against drought and heat stress than diploid *C. rubens*, traits that will benefit the cultivation of these plants, particularly in rain-fed cropping systems.

Lu et al. (2022) in contrast, explored the importance of the bZIP family in tea plants, and how this protein family plays a vital role in various biological processes, including seed maturation, flower development, light signal transduction, pathogen defence, and various stress responses. Specifically, they identified a total of 76 CsbZIP genes from the tea plant genome. Phylogenetic analysis with Arabidopsis counterparts revealed that CsbZIP proteins clustered into 13 subgroups, among which 13 ABFs were related to the ABA signalling transduction pathway. Transcriptome analysis revealed the expression profiles of CsABF genes in different tissues (bud, young leaf, mature leaf, old leaf, stem, root, flower, and fruit) and under diverse environmental stresses (drought, salt, chilling, and MeJA).

Ultimately, concerted efforts are needed to advance the research and development of orphan crop species so that food security will be strengthened, and livelihoods enhanced. To this end, advances in low-cost spectral phenotyping, genomics, genomic selection, and artificial intelligence, are giving powerful resources to plant breeders and plant scientists, greatly accelerating the genetic gains for orphan crop improvement.

## Author contributions

BC (Conceptualization, Writing, Review & Editing), DD (Conceptualization, Writing, Review & Editing), WL (Review & Editing) & LH (Review & Editing). All authors contributed to the article and approved the submitted version.

